# Stressful life events and cancer risk

**DOI:** 10.1038/sj.bjc.6603471

**Published:** 2006-11-14

**Authors:** C Bergelt, E Prescott, M Grønbæk, U Koch, C Johansen

**Affiliations:** 1 Danish Cancer Society, Institute of Cancer Epidemiology, 2100 Copenhagen, Denmark; 2 Institute of Medical Psychology, University Clinic Hamburg, 20246 Hamburg, Germany; 3 Rigshospitalet, Department of Cardiology, 2100 Copenhagen, Denmark; 4 The Copenhagen City Heart Study, Bispebjerg Hospital, 2400 Copenhagen, Denmark; 5 National Institute of Public Health, 1399 Copenhagen, Denmark

**Keywords:** epidemiology, neoplasms, cohort study, stressful life events, stress

## Abstract

In a prospective cohort study in Denmark of 8736 randomly selected people, no evidence was found among 1011 subjects who developed cancer that self-reported stressful major life events had increased their risk for cancer.

The assumption of an association between stress and cancer occurrence is popular in the lay public (Baghurst *et al*, 1992) and among cancer patients (Stewart *et al*, 2001) and the topic has received intensive research attention in the past. Positive associations between severe stressors (i.e., stressful life events) and cancer risk have frequently been reported from retrospective and case–control studies, whereas prospective studies with unbiased data sources for exposure assessment, such as administrative registers, have shown no association between stressors, like death of a spouse, divorce, death of a child or serious illness in a child, and the subsequent risk for cancer (Johansen and Olsen, 1997; Kvikstad *et al*, 1994).

We investigated the association between self-reported experience of stressful life events and the risk for cancer. As opposed to many previous prospective studies (Johansen and Olsen, 1997; Li *et al*, 2002; Dalton *et al*, 2004), all analyses were adjusted for a number of lifestyle factors known to be associated with cancer risk.

## MATERIALS AND METHODS

Data were obtained from the Copenhagen City Heart Study (Appleyard *et al*, 1989; Schnohr *et al*, 2001), a population-based, randomly sampled cohort study which was initiated in 1976. The data for the study reported here stem from the third survey (1991–1994), when a total of 10 135 people completed a questionnaire and attended a health examination (response rate, 61%). A cancer diagnosis before date of entry resulted in the exclusion of 589 people (6%) from the analysis. A further 810 (8%) were excluded because of missing data on at least one of the included variables, leaving 8736 (86%) eligible for the data analyses.

Follow-up for cancer occurrence began on the date of the examination between 10 October 1991 and 16 September 1994, and ended on the date of diagnosis of a first primary cancer, emigration, death or 30 November 2002, whichever came first. Cancer cases were ascertained by linkage with the Danish Cancer Registry, which has registered all cases of malignant neoplasms in Denmark in a population-based system since 1942 (Jensen *et al*, 1985). Comprehensive evaluation has shown that the Danish Cancer Registry is 95–99% complete and valid (Storm *et al*, 1997).

Following the question ‘Did you ever experience one of the following events?’, all people completed a checklist of 12 stressful events. Five of these were job-related (job loss, prospect of promotion which never occurred, long-lasting or serious conflicts with colleagues, superiors or subordinates) and seven events referred to family and adult life (long-lasting or serious illness of a child, educational problems with children, conflicts with adult children, marital problems, personal illness or accidents, illness or death of a family member, economic problems). For the analyses, a sum score was calculated, and the sample was classified into four groups with regard to the number of types of event experienced (no, one, two and three or more types of event).

The analyses of the relation between cancer incidence rates and stressful life events were based on sex stratified Cox proportional hazard models with age as the time axis to ensure that the estimation procedure was based on comparisons of individuals at the same age. Time under study was included as a time-dependent variable and was modelled by a linear spline with boundaries at 1, 2 and 3 years after entry into the study. A linear spline was used because this allows the hazard to be nonlinear (Greenland, 1995a). All models were adjusted for physical activity (sedentary, moderately active, active), drinking status (nondrinkers *vs* consumers), alcohol intake (drinks per day), smoking status (never, former and current smokers of 1–14, 15–24 and ⩾25 g of tobacco per day), duration of smoking, body mass index, school education (<8 years, 8–11 years, >11 years), household income (<100 000; 100 000–149 000; 150 000–199 000; 200 000–299 000, 300 000–399 000 and ⩾400 000 DKK) and cohabitation status (living alone *vs* not living alone). Associations were estimated for the two sexes separately and for the two sexes combined. In the combined analysis, we did not find a significant difference with regard to life events between the two sexes, using the Wald test. All analyses were stratified according to sex such that the basic (underlying) hazards were sex-specific.

Whenever possible, variables were entered linearly into the Cox model, because this is biologically more reasonable than the step functions corresponding to categorisation and, furthermore, increases the power of the analyses (Greenland, 1995b). The linearity of the associations was evaluated graphically by linear splines with three boundaries (Greenland, 1995a) placed at the quartiles among cases. Body mass index and daily alcohol consumption could be entered as linear variables.

Two-sided 95% confidence intervals (CIs) for the hazard ratio (HR) were calculated from Wald's test of the Cox regression parameter on the log rate ratio scale. The PHREG procedure in SAS (release 8.2; SAS Institute Inc., Cary, NC, USA) on the TextPad platform was used for statistical analyses.

## RESULTS

The most frequent type of event reported by both sexes was ‘long-lasting or serious illness or death of a family member’ (women: 55%; men: 40%); the second most frequent was ‘long-lasting or serious marital problems’ for women (23%) and ‘job loss’ for men (23%). In general, men reported job-related stressful life events more frequently than women, and women reported stressful events related to family and adult life more frequently than men (data not shown).

The median follow-up time was 9.3 years (range, 0–11.2 years). During this time, cancer was diagnosed in 1011 people. Those who reported three or more types of stressful life event were significantly younger than the rest of the sample and had significantly poorer health behaviour with regard to alcohol and tobacco consumption at baseline. Further, this group included significantly more people reporting long schooling and living alone (Table 1). However, the number of different types of stressful life event experienced was not associated with cancer risk (Table 2).

## DISCUSSION

The analyses show that the accumulated experience of stressful life events is associated with an unhealthy lifestyle but is not associated with an increase in cancer incidence. The results of this large, prospective, population-based study therefore do not support the hypothesis that life stress, when defined as stressful life events, increases the risk for developing cancer.

Despite the prospective design, the study also has some limitations. People who experienced stressful events in the past and subsequently developed cancer, but before date of entry into the study cohort, were excluded from the analyses, whereas those who experienced events at a similar time and did not develop cancer remained in the analyses. The analyses may therefore have been biased and thus underestimate the true association. However, we had access to information on incident cases of cancer among cohort members back to 1942. Furthermore, people indicated whether they had experienced one or more of the events on the checklist, but there was no means of ascertaining whether the event had been experienced only once or several times. Therefore, some people may have been misclassified. Nevertheless, there is no reason to assume that the frequency of repeated events was unequally distributed between groups, and it can thus be assumed that the average experience of stressful events increased in groups in ascending order.

Our results accord with previous studies by our group, which utilised administrative sources for the assessment of exposure and outcome. In none did we find evidence for an independent link between psychological factors such as severe depression (Dalton *et al*, 2002), personality traits (Hansen *et al*, 2005) or stressful life events (Li *et al*, 2002; Dalton *et al*, 2004) and cancer risk. We conclude that there is no convincing evidence that psychological factors cause cancer.

## Figures and Tables

**Table 1:** Sample characteristics at date of entry of 8736 persons in the third survey of the Copenhagen City Heart Study (1991–2002), Denmark

	**No. of types of stressful life event experienced**				
**Characteristic**	**0** ***n*=2349**	**1** ***n*=2628**	**2** ***n*=1783**	**⩾3** ***n*=1976**	***P* a**
Age in years (mean, s.d.)	59.4 (16.2)	58.3 (15.5)	56.6 (15.1)	54.5 (13.4)	<0.001
Alcohol consumption in drinks per day (mean, s.d.)	0.7 (0.9)	0.8 (1.0)	0.8 (1.0)	1.0 (1.3)	<0.001
Body mass index (mean, s.d.)	25.7 (4.3)	25.7 (4.3)	25.4 (4.3)	25.5 (4.4)	0.167
Physically inactive (%)	13	11	11	13	0.125
*Tobacco consumption*					
Never smoked (%)	29	27	25	21	< 0.001
Current smoker > 25 g per day (%)	6	6	7	11	< 0.001
Living alone (%)	29	39	40	42	< 0.001
School education >11 years (%)	22	23	26	27	< 0.001
Low household income (%)	20	19	19	22	0.064

a Two-sided *P*-values calculated from analysis of variance or *χ*^2^.

**Table 2:** Cancer risk by number of types of stressful life event experienced by 8736 people in the third survey of the Copenhagen City Heart Study (1991–2002), Denmark

**No. of types of stressful life event experienced**	**Cases/*n***	**HR (95% CI)^a^**
None^b^	302/2349	1.0
1	308/2628	0.95 (0.81–1.11)
2	187/1783	0.89 (0.74–1.08)
3 or more	214/1907	0.99 (0.82–1.19)

CIs=confidence intervals; HR=hazard ratio.

a Adjusted for behavioral (physical activity during leisure time, tobacco consumption, duration of smoking, alcohol abstinence, daily alcohol consumption, body mass index) and socioeconomic factors (years of schooling, household income, cohabitation).

b Reference category.
